# Evaluation of Alpha1 Antitrypsin Deficiency-Associated Mutations in People with Cystic Fibrosis

**DOI:** 10.3390/jcm14196789

**Published:** 2025-09-25

**Authors:** Jose Luis Lopez-Campos, Pedro García Tamayo, Maria Victoria Girón, Isabel Delgado-Pecellín, Gabriel Olveira, Laura Carrasco, Rocío Reinoso-Arija, Casilda Olveira, Esther Quintana-Gallego

**Affiliations:** 1 Unidad Médico-Quirúrgica de Enfermedades Respiratorias, Instituto de Biomedicina de Sevilla (IBiS), Hospital Universitario Virgen del Rocío/Universidad de Sevilla, 41013 Seville, Spain; pfgartam@gmail.com (P.G.T.); lauracarrascohdez@gmail.com (L.C.); rocioreari@gmail.com (R.R.-A.); esther.quintana@telefonica.net (E.Q.-G.); 2 Centro de Investigación Biomédica en Red de Enfermedades Respiratorias (CIBERES), Instituto de Salud Carlos III, 28029 Madrid, Spain; idelpe@gmail.com; 3 Servicio de Neumología, Hospital Regional Universitario de Málaga, 29010 Málaga, Spain; marivi_giron@hotmail.com (M.V.G.); casi1547@separ.es (C.O.); 4 Departamento de Medicina y Dermatología, Universidad de Málaga, 29071 Málaga, Spain; gabrielm.olveira.sspa@juntadeandalucia.es; 5 Instituto de Investigación Biomédica de Málaga (IBIMA)-Plataforma Bionand, 29009 Málaga, Spain; 6 Servicio de Pediatría, Hospital Universitario Virgen del Rocío, 41013 Seville, Spain; 7 Departamento de Farmacología, Pediatría y Radiología, Universidad de Sevilla, 41009 Seville, Spain; 8 UGC Endocrinología y Nutrición, Hospital Regional Universitario de Málaga, IBIMA Plataforma BIONAND, 29009 Málaga, Spain; 9 Centro de Investigación Biomédica en Red de Diabetes y Enfermedades Metabólicas Asociadas (CIBERDEM), Instituto de Salud Carlos III, 28029 Madrid, Spain

**Keywords:** alpha1 antitrypsin deficiency, cystic fibrosis, genetic studies, prevalence, screening

## Abstract

**Background**: Recent hypotheses suggest that mutations associated with alpha1 antitrypsin (AAT) deficiency (AATD) may influence the clinical presentation and progression of cystic fibrosis (CF). This study employs a longitudinal design to determine the prevalence of AATD mutations and assess their impact on CF. **Methods**: The study Finding AAT Deficiency in Obstructive Lung Diseases: Cystic Fibrosis (FADO-CF) is a retrospective cohort study evaluating people with CF from November 2020 to February 2024. On the date of inclusion, serum levels of AAT were measured and a genotyping of 14 mutations associated with AATD was performed. Historical information, including data on exacerbations, microbiological sputum isolations, and lung function, was obtained from the medical records, aiming at a temporal lag of 10 years. **Results**: The sample consisted of 369 people with CF (40.9% pediatrics). Of these, 58 (15.7%) cases presented at least one AATD mutation. The AATD allelic combinations identified were PI*MS in 47 (12.7%) cases, PI*MZ in 5 (1.4%) cases, PI*SS in 3 (0.8%) cases, PI*SZ in 2 (0.5%) cases, and PI*M/P_lowell_ in 1 (0.3%) case. The optimal cutoff value for AAT levels to detect AATD-associated mutation carriers was 129 mg/dL in the overall cohort (sensitivity of 73.0%; specificity 69.2%) and 99.5 mg/dL when excluding PI*MS cases (sensitivity 98.0%; specificity 90.9%), highlighting the need for lower thresholds in clinically severe genotypes to improve case detection. The number of mild exacerbations during the follow-up appeared to be associated with AATD mutations. **Conclusions**: AATD mutations are prevalent in CF and may impact certain clinical outcomes. If systematic screening was to be planned, we recommend considering the proposed cut-off points to select the population for genetic studies.

## 1. Introduction

Alpha1 antitrypsin deficiency (AATD) is a rare genetic disorder caused by mutations in the SERPINA1 gene. This condition, often underdiagnosed [1,2], can potentially impact the hepato-pulmonary system and significantly affect patients suffering from its severe forms. The presence of AATD may be particularly relevant in clinical situations where there is an absence of proteolytic counterbalance. This scenario has been described for COPD [3], but it may also be relevant in other clinical situations. Notably, AATD could be particularly detrimental in diseases that result in increased elastolytic capacity [4], such as bronchiectasis and cystic fibrosis (CF).

CF is another rare genetic disorder caused by mutations in the cystic fibrosis transmembrane conductance regulator (CFTR) gene. This disease has a significant clinical impact on patients and their families and currently presents several challenges. Specifically, the evaluation of the impact of various comorbidities and distinct phenotypic manifestations that might influence the clinical presentation or disease progression [5] are areas of interest for exploration [6]. In this context, it has been hypothesized that SERPINA1 mutations might have a clinical impact on CF [4,7].

The relationship between CF and AATD has been explored in various studies [8,9,10,11,12]. Collectively, these studies describe the prevalence of SERPINA1 mutations in people with CF (pwCF) and evaluate their potential clinical impact. However, these studies limit their analysis to a small number of CFTR and SERPINA1 mutations, and some do not have a long follow-up. Therefore, a new analysis is needed to describe the prevalence and longitudinally evaluate the impact of different SERPINA1 mutations on pwCF. The aim of the present study is to build upon previous analyses of the influence of AATD on CF disease progression. By using a longitudinal design and including all mutations related to both conditions in a mixed pediatric-adult population, we aim to provide updated information on the potential influence of SERPINA1 mutations on the presentation and progression of CF.

## 2. Materials and Methods

The study *Finding AAT Deficiency in Obstructive Lung Diseases: Cystic Fibrosis* (FADO-CF) was a retrospective cohort study. It evaluated people with a confirmed diagnosis of CF in an outpatient setting at two university hospitals in southern Spain. All adult and pediatric cases evaluated during routine visits for CF from November 2020 to February 2024 were invited to participate. The sole exclusion criterion was the patient’s or their guardian’s unwillingness to participate or their experiencing an exacerbation on the inclusion date. In such cases, inclusion was postponed until the next visit. Medical records were obtained from clinical information reflected in their medical history and from data obtained directly from the participants. Cases under 18 years of age were considered pediatric cases. The study received approval from our institution’s ethical committee (approval act 18/2020) and adhered to the recommendations of the Helsinki Declaration for research involving human subjects, as well as current Spanish and European legislation. All participants or their legal guardians provided written informed consent prior to being included in the study.

The index date was the date of inclusion. During this visit, we performed serum determinations of AAT and C-reactive protein (CRP), along with genotyping of AAT mutations, as reported below. We also recorded the spirometry closest to the index date when the patient was not experiencing an exacerbation. High-resolution computed tomography scans closest to the index date were obtained. The classification of radiological involvement was done using the Bhalla scoring system [13]. Additionally, the number of affected lobes was noted using the following classification: no bronchiectasis, one lobe, different lobes in one hemithorax, bilateral (3 or fewer lobes), diffuse (4 or more lobes affected).

Historical information was obtained from medical records, aiming at a temporal lag of 10 years. During this time, the total number of exacerbations, the first available pulmonary function test, and sputum microbiological isolations were obtained from the medical record. Exacerbations were manually recorded by exhaustively reviewing all the notes of the clinical evolution over the follow-up. Exacerbations were considered severe if they resulted in hospital admission or required intravenous therapy at home. Any exacerbations that required oral treatment only were considered mild. Sputum studies were performed by our central microbiology laboratory using conventional culture techniques for bacteria and fungi. A chronic bronchial infection was defined, according to current recommendations [14] as two or more isolates of the same organism at least 3 months apart in 1 year. The absolute values obtained in spirometry were expressed in liters and as a percentage of their predicted value. The predicted values were calculated according to the equations of the Global Lung Function Initiative [15]. With current spirometry and the earliest available spirometry during follow-up we estimated the annualized forced expiratory volume in one second (FEV_1_) decline expressed as mL/year for those cases with at least 5 years of follow-up.

### 2.1. Laboratory Tests

Allele-specific genotyping was conducted on the index date using the Progenika A1AT Genotyping Test (Progenika Biopharma SA, Zamudio, Vizcaya, Spain). This test employs polymerase chain reaction amplification to generate large quantities of the target sequences in the SERPINA1 gene. The Luminex^®^ 200 system is then used to detect the previously labeled amplified fragments, as described earlier [16]. The test is designed for use with genomic DNA extracted from human whole blood samples collected in K3-ethylenediaminetetraacetic acid tubes, dried blood spots, or human buccal swab samples. For the current analysis, we used human buccal swab samples collected with the OCR100 ORAcollect-DNA collection kit. Both the test and the OCR100 buccal swab used for sample collection are CE marked (European Conformity) and approved by the United States Food and Drug Administration.

The test can identify the 14 most common deficiency variants of the SERPINA1 gene, namely PI*S, PI*Z, PI*I, PI*M_procida_, PI*M_malton_, PI*S_iiyama_, PI*Q0_granite falls_, PI*Q0_west_, PI*Q0_bellingham_, PI*F, PI*P_lowell_, PI*Q0_mattawa_, PI*Q0_clayton_, and PI*M_heerlen_. If none of the 14 alleles studied were found, the result was noted as negative and interpreted as an M allele, since the absence of any of these 14 alleles suggests with over 99% probability that the genotype corresponds to PI*M. SERPINA1 gene sequencing was conducted when none of the 14 mutations were found or when one variant was detected in heterozygous status and the AAT serum level was less than 60 mg/dL, or when requested by the clinician in charge.

Serum AAT and CRP were measured as part of our AATD protocol [17] using nephelometry with the Atellica NEPH 630 System (Siemens Healthineers AG, Forchheim, Germany). The normal range for AAT was defined as 90 to 200 mg/dL. A serum value falling below 57.2 mg/dL was interpreted as the protective threshold, indicative of severe AATD. CRP values above 5 mg/L were considered elevated, but values above 20 mg/L were considered to potentially significantly influence AAT concentrations [18]. Additionally, hepatic impairment was assessed by collecting the most recent available values of total bilirubin, alkaline phosphatase, alanine transaminase, aspartate transaminase, albumin, prothrombin time, and platelet count.

### 2.2. Data Processing and Analysis

Data processing was conducted using IBM SPSS Statistics software, version 29.0 (IBM Corporation, Armonk, NY, USA). A *p*-value of 0.05 was considered statistically significant. Variables were described using either the mean and standard deviation (in parentheses) or absolute and relative frequencies (in parentheses), depending on the nature of the variables.

First, we described the frequency of AATD alleles in our population. Then, the cutoff point for AAT values associated with carrying an AATD-related allele was determined by constructing receiver operating characteristic (ROC) curves. Sensitivity and specificity were estimated for each value on the curve, with the cutoff point chosen as the one with the highest Youden index. Finally, the sensitivity and specificity of the indicated cutoff point were calculated, along with their 95% confidence intervals.

Afterwards, to compare cases with and without SERPINA1 mutations, we employed the Chi-square test (or Fisher’s exact test) for categorical variables and Student’s *t*-test for independent data. The latter was used after verifying the homogeneity of variances with Levene’s test for continuous variables. Those significant differences and those close to significancy (*p* < 0.1) were further explored with a binomial multivariate logistic regression analysis using the presence of any AATD mutation as the dependent variable, and gender, age, F508del carriers and the use of CFTR modulators as confounders. The results were expressed in odds ratios with the 95% confidence intervals. To compare the decline in FEV1 between the two groups (with and without AATD mutation) we used density plots. A Density Plot visualizes the distribution of data over a continuous interval or time period. This chart is a variation of a Histogram that uses kernel smoothing to plot values, allowing for smoother distributions by smoothing out the noise. The peaks of a Density Plot help display where values are concentrated over the interval.

Finally, we also evaluated the differences in the studied parameters by separating patients with the PI*MS genotype from the rest. Consequently, in a second analysis, we created three genotype groups: PI*MM, PI*MS, and the remaining genotypes. This comparison was performed using one-way analysis of variance (ANOVA), after verifying the equality of variances with Levene’s test. If homoscedasticity was not met, we conducted Welch’s test. Intergroup differences were explored with the Bonferroni corrections for those found significantly different between groups. Similarly, those significant differences and those close to significancy (*p* < 0.1) were further explored with a binomial multivariate logistic regression analysis using the presence of any AATD mutation (excluding PI*MS) as the dependent variable, and gender, age, F508del carriers and the use of CFTR modulators as confounders. The results were also expressed in odds ratios with the 95% confidence intervals.

## 3. Results

The study sample comprised 369 pwCF, of which 151 (40.9%) were pediatric cases, 176 (47.7%) were males, and 278 (75.3%) were carriers of at least one CFTR F508del mutation. A summary of the cohort description is provided in Table 1. A subset of 28 (7.5%) cases was incorporated at the time of diagnosis by our units; hence, they had no prior follow-up and were only available for the initial visit. The retrospective follow-up period of the resting 341 cases, spanning from the CF diagnosis to the AlphaID evaluation, averaged 16.3 years with a standard deviation of 11.4 years.

### 3.1. Prevalence of AAT Alleles

**Figure 1:**
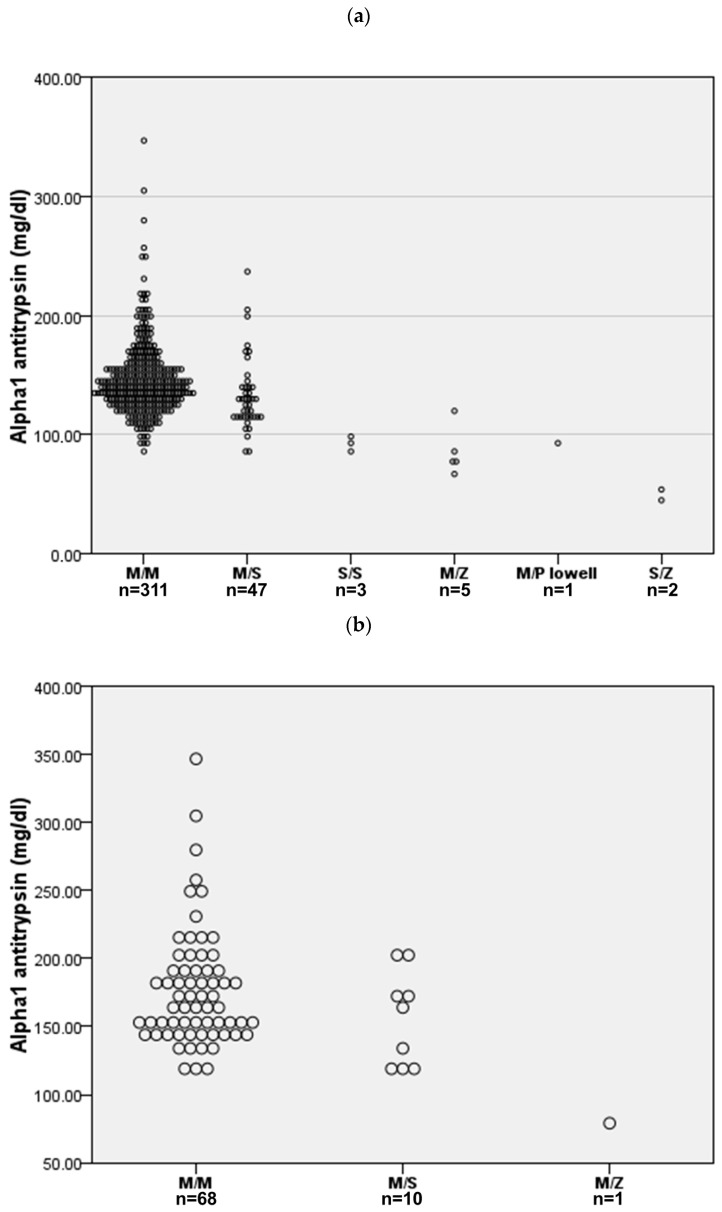
AAT serum concentration for the different allele combinations. (**a**) All cases (n = 369). (**b**) Cases with C-reactive protein above 5 mg/L (n = 79).

A mutation associated with AATD was found in 58 (15.7%) cases. The AATD allelic combinations identified were PI*MS in 47 (12.7%) cases, PI*MZ in 5 (1.4%) cases, PI*SS in 3 (0.8%) cases, PI*SZ in 2 (0.5%) cases, and PI*M/P_lowell_ in 1 (0.3%) case. Serum AAT levels by allele combinations are shown in Figure 1a. Only the two SZ cases presented a serum AAT below the protective threshold. Interestingly, 79 (21.4%) cases presented an increased CRP above 5 mg/L, and 22 (6.0%) were above 20 mg/L. As expected, AAT levels in those cases with elevated CRP were significantly increased for both 5 mg/L and 20 mg/L thresholds, but the proportion of AAT mutations was similar (Figure 1b).

**Figure 2:**
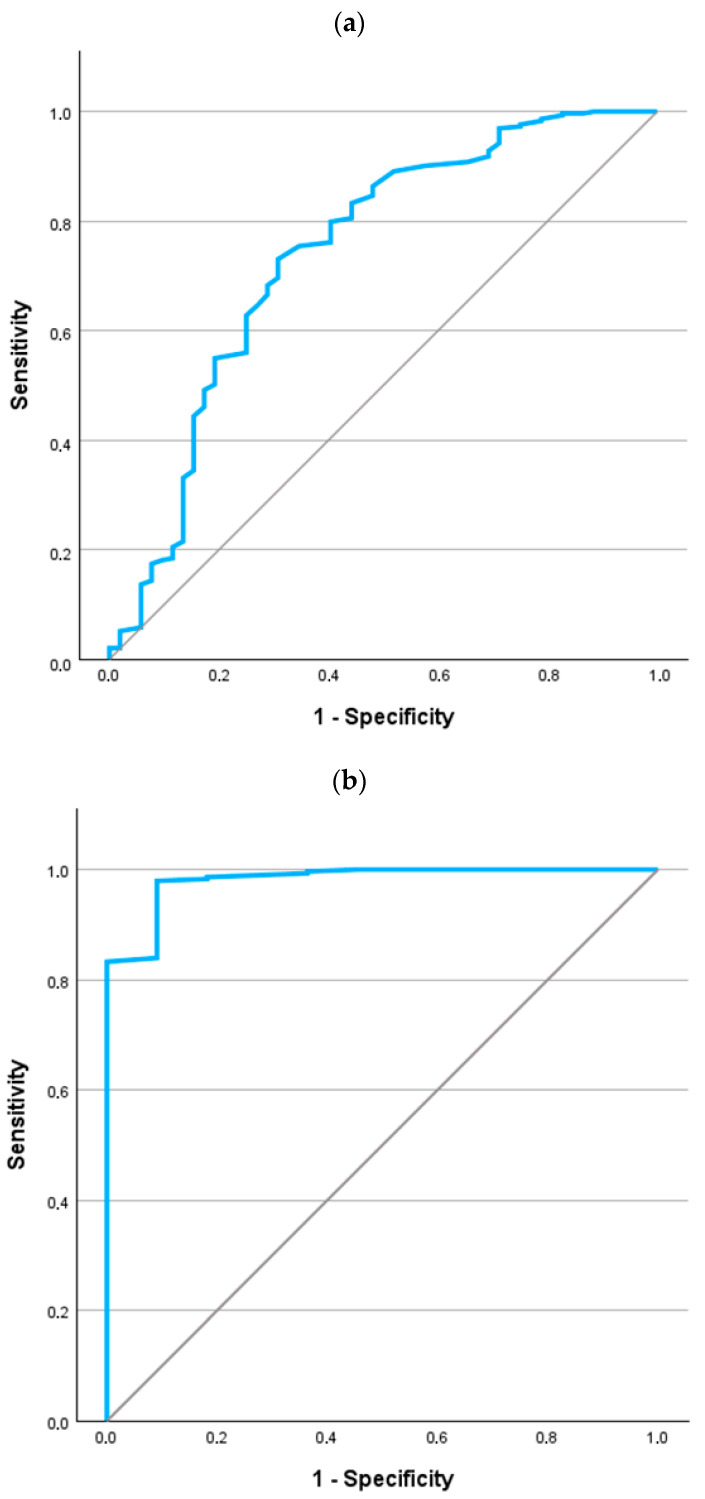
ROC curves for the values of AAT in the identification of AATD in patients with CF. (**a**) All cases (n = 369). (**b**) Excluding PI*MS cases (n = 322).

The ROC curve for a diagnosis of AATD in CF is referenced in Figure 2. For the entire population (Figure 2a), the area under the curve was 0.740 (*p* < 0.001; 95% confidence interval 0.658 to 0.823). For the detection of any AAT mutation, the best cutoff value was 129 mg/dL. This reported a sensitivity of 73.0% (95% confidence interval 68.4% to 77.7%) and specificity of 69.2% (95% confidence interval 64.4% to 74.1%).

If we focus the analysis on evaluating the potentially more severe mutation (i.e., eliminating cases with the PI*MS genotype), the ROC curve is shown in Figure 2b. The area under the curve was 0.981 (*p* < 0.001; 95% confidence interval 0.953 to 1.000). For the detection of any AAT mutation, the best cutoff value was 99.5 mg/dL. This reported a sensitivity of 98.0% (95% confidence interval 96.4% to 99.5%) and specificity of 90.9% (95% confidence interval 87.7% to 94.1%).

### 3.2. Association of AAT Mutations with CF Clinical Outcomes

**Figure 3:**
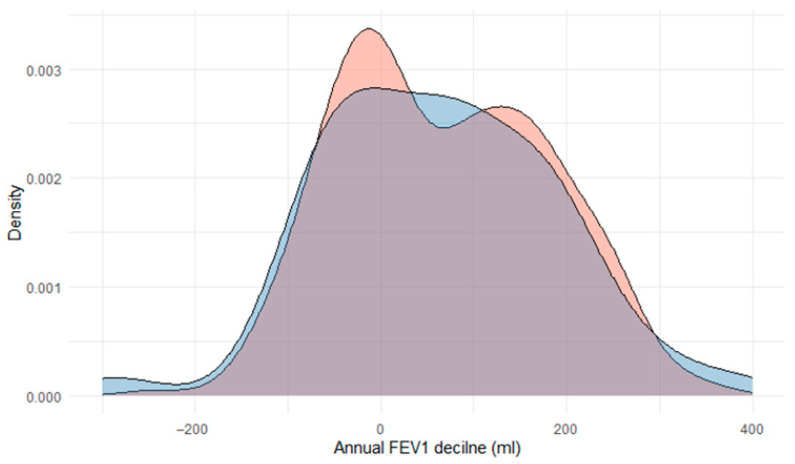
Density plot of annual FEV_1_ decline in those cases carrying an AAT mutation (red; n = 58) and those without (blue; n = 311).

**Lung function.** A crude bivariate comparison of FEV_1_ on the index date between cases carrying any SERPINA1 mutation versus the rest of the cohort showed a trend towards a worsening for those carrying any AATD mutation (Table 1). However, these differences disappeared in the multivariate analysis (Supplementary Table S1). Of note, after excluding subjects with the PI*MS mutation, differences in FEV1 persisted in the multivariate analysis (Supplementary Table S1). The annualized FEV_1_ decline for those 201 cases (54.5%) with at least 5 years of follow-up was not significantly different between groups (Figure 3). This result is especially revealing, as it relates to one of the key prognostic markers for disease progression. When analyzing the cases divided into three groups (PI*MM, PI*MS, and the remaining mutations), we found no differences among these three groups in any of the functional variables.

**Exacerbation frequency.** CF cases carrying any SERPINA1 mutation showed a numerical increase in the number of mild exacerbations close to significance (10.7 (9.7) vs. 13.1 (12.1); *p* = 0.057), with no difference in severe exacerbations (2.1 (4.1) vs. 2.3 (3.1); *p* = 0.415) over the follow-up. The difference in mild exacerbations just reached significance in the multivariate analysis with an odds ratio of 1.028 (95% confidence interval: 1.000 to 1.056; *p* = 0.049). The occurrence of exacerbations in CF is currently recognized as one of the main prognostic markers of disease progression. The time elapsed between the diagnosis of CF and the first mild or severe exacerbation was not significantly different in both groups (Supplementary Figure S1). When analyzing the cases divided into three groups (PI*MM, PI*MS, and the remaining mutations), we found no differences among these three groups in any of the exacerbation endpoints.

**Radiological involvement.** The degree of radiological involvement was not different in cases with any SERPINA1 mutation. The number of lobes affected was equally distributed between both groups (3.5 (2.4) vs. 3.2 (2.1) lobes; *p* = 0.744). The Bhalla classification system was also similar in both groups, with 17.7 (6.4) points for non-SERPINA1 carriers and 18.1 (6.7) points for those carrying any SERPINA1 mutation (*p* = 0.318). When analyzing the cases divided into three groups (PIMM, PIMS, and the remaining mutations), we found a numerical decrease in the Bhalla punctuation with PI*MM 17.7 (6.4), PI*MS 18.8 (6.6) and other genotypes 15.8 (6.5) points, although the difference was not significant. The number of affected lobes was also numerically different with PI*MM 2.8 (2.4), PI*MS 2.0 (2.1) and other genotypes 3.1 (2.5) lobes. This difference was close to significance (*p* = 0.091) but disappeared after the multivariate analysis.

**Chronic bronchial infection.** In the analysis, 220 (59.6%) cases presented a chronic bronchial infection. The microorganisms responsible for this chronic bronchial infection were *Pseudomonas aeruginosa* in 81 (22.0%) cases, *Staphylococcus aureus* in 125 (33.9%) cases, and other microorganisms in 21 (5.7%) cases, including *Burkholderia cepacea*, *Alcalygenes xylosoxidans*, *Haemophilus influenzae*, and fungi. In 12 (3.3%) cases, two different microorganisms were isolated in the sputum of the same patient. The percentage of cases with chronic bronchial infection was similar (*p* = 0.645) for both carriers (33 cases; 56.9%) and non-carriers of SERPINA1 mutations (187 cases; 60.1%). All the 12 cases with the isolation of two microorganisms were non-carriers of SERPINA1 mutations. The time elapsed from birth to the first isolation was not significantly different between groups (11.8 (10.4) years for non-carriers vs. 13.1 (14.2) years for carriers; *p* = 0.338). When analyzing the cases divided into three groups (PI*MM, PI*MS, and the remaining mutations), we found no differences among these three groups in any of the chronic bronchial infection endpoints.

**Lung and liver transplantation.** In the complete cohort, 44 (11.9%) had undergone a lung transplantation and there were 4 (1.1%) cases of liver transplantation. The proportion of people with a lung transplantation was not different between groups (35 (11.3%) cases for non-carriers vs. 9 (15.5%) for carriers; *p* = 0.358). None of the 4 cases with liver transplantation were carriers of AAT mutations. The biochemical and hematological parameters of hepatic impairment were similar between patients with and without mutations associated with AATD (Supplementary Table S2). The analysis of the cases divided into three groups (PI*MM, PI*MS, and the remaining mutations) showed no differences among these three groups in any of the biochemical and hematological parameters of hepatic impairment.

## 4. Discussion

This study is among the few that have evaluated the relationship between two distinct rare conditions. Our findings indicate that (1) AAT mutations are prevalent in pwCF; (2) we have established optimal cutoff values for screening AATD in pwCF; (3) the impact of carrying AAT mutations did not significantly influence most of the clinical outcomes, except for a potential impact on lung function and mild exacerbations.

AATD is a unique genetic condition in terms of its inheritance pattern. Its most severe mutations are associated with respiratory and liver diseases, but with a highly variable penetrance that is largely dependent on the allelic combination of each case Interestingly, the mechanisms of this differential effect are not yet fully understood. This situation becomes even more complex when we focus on diseases other than COPD, much less explored in the literature. Some of the most controversial examples are its relationship to the clinical presentation and control of bronchial asthma [19,20] and its potential impact on viral infections [21]. In this context, the relationship of AATD with bronchiectasis has been explored to the point that screening for AATD in patients with bronchiectasis is currently recommended. However, the potential importance of AATD in pwCF has been less consistently explored. The present study completes and updates the relationship between these two rare diseases.

This study combines the analysis of a large population of pwCF from two referral units, together with the systematic genotyping of all the cases with a comprehensive genetic method that has proven effective in real life in several countries, ensuring a correct diagnosis of AATD in this population. However, it is necessary to keep in mind some methodological considerations in order to put our results in the proper context. First of all, it is a retrospective longitudinal follow-up. Of note, our clinics are organized in such a way that data collection is very systematic, and everything is conveniently recorded in the clinical history. Secondly, some cases were included at the time of diagnosis, so they did not have retrospective follow-up. Thirdly, the alleles found in relation to AATD were limited, as befits a rare genetic condition. In particular, there were no PI*ZZ cases in our cohort and only two PI*SZ patients, making the exploration of these more severe genotypes uncertain. It is true that we obtained at least 3 cases of PI*SS. In this regard, the PI*SS has been recently shown to be as pathogenetic as PI*SZ [22]. Fourthly, ours was a cohort solely composed of pwCF with no control groups. Additionally, this study was done in Spain, a country with a higher prevalence of SERPINA1 alleles [23,24]. Therefore, our prevalence findings may not be similar to other world regions. Fifth, about half of our participants were receiving CFTR modulators therapy, which might impact on the progression of the disease and the clinical outcomes evaluated [25,26]. Sixth, as these drugs are dispensed exclusively through hospital pharmacies under direct supervision, treatment adherence was assessed based on the direct dispensing records from the hospital pharmacy. Follow-up was performed by the pharmacy team, with alerts generated in cases of missed collections. However, no questionnaires or electronic monitoring of medication intake were included in the study protocol. Ultimately, both CF and AATD can impact liver function. Consequently, it is anticipated that the co-occurrence of these conditions may result in a more severe manifestation of liver disease [27]. However, only four cases in our cohort underwent liver transplantation, rendering the sample size insufficient to adequately assess this outcome. Finally, data related to the transplantation process were not collected in the present study as part of our analysis.

Our report informs on the prevalence of AATD alleles in a CF population. Only a few studies have explored the prevalence of SERPINA1 mutations in pwCF (Table 2). The first study to our knowledge was done in Germany in a pediatric population exploring 215 children with CF and 752 healthy individuals [8]. The authors studied AATD phenotypes by isoelectric focusing and found 17 (7.9%) of PI*MS, 1 (0.4%) PI*SS and 10 (4.6%) PI*MZ. Later, in the United Kingdom a group of 79 children who had undergone lung transplantation or had died from pulmonary disease had SERPINA1 mutations explored in blood spots [9]. The authors explored the presence of the PI*Z and PI*S alleles exclusively and found 1 (1.2%) PI*MZ case and 4 (5.0%) cases of PI*MS. A more complete study from the same group including 157 unrelated adults and children with CF reported that same year 16 (10.1%) PI*MS, 1 (0.6%) PI*SS and 3 (1.9%) PI*MZ out of 147, by phenotyping. More recently, 269 pwCF from Southern Germany homozygous or heterozygous for the major CFTR mutation F508del were included in another study [11] and found 16 cases carrying PI*MS (6%) and 5 cases carrying PI*MZ (1.9%). A multicenter Canadian study evaluated 716 pwCF and were genotyped for the PI*Z and the PI*S alleles [12]. These authors found 69 (9.6%) PI*MS, 13 (1.8%) PI*SS and 18 (2.5%) PI*MZ cases. Finally, in Italy a recent study reported the results of genotyping 173 pwCF finding only 9 (5.2%) PI*MS with no other SERPINA1 alleles found [28]. Altogether, our results are in line with these previous findings but with an increase in the number of PI*MS cases and the finding of two PI*SZ cases. These studies were carried out many decades ago when not as many mutations of AATD were known, with more limited diagnostic tools and they explored people with specific CF mutations. Interestingly, no previous report has shown the clinical behavior of CF + PI*ZZ neither in the literature nor in our study. These results are particularly relevant given the relatively low prevalence of the PI*S and PI*Z alleles in the general population [29,30]. Specifically, the frequency of the PI*S allele ranges from 7.3 to 31.3 per 1000 individuals in Northern Europe, and from 18.6 to 185.1 per 1000 in Western, Southern, and Central Europe. Similarly, the PI*Z allele ranges from 0.0 to 45.1 per 1000 in Northern Europe, and from 7.3 to 29.7 per 1000 in Western, Southern, and Central Europe [24]. These values are notably lower than those observed in studies assessing AATD prevalence among individuals with CF. This suggests that patients with CF may represent a population with an increased prevalence of AATD-related mutations.

An intriguing observation is the differential gender distribution between the two AATD groups, with a higher prevalence of women in the group harboring any AATD mutation. The significance of gender in the clinical manifestation of COPD is well-documented; however, its impact on AATD patients remains underexplored. Analysis of the German AATD registry indicates that females may experience symptomatic disease with a lesser history of tobacco use and face a more delayed diagnosis [31]. Similarly, in CF, gender has been reported to influence disease progression and clinical outcomes [32]. Consequently, to account for its potential confounding effect, we included gender as a covariate in the multivariate models.

The clinical impact of SERPINA1 mutations on CF progression was not very striking. After all the outcomes were evaluated, in our study we only observed some difference in the number of mild exacerbations. Reviewing previous studies, only one study in pediatric cases found an earlier time to first microbiological isolation and another study found a relationship with lung function but in the reverse, i.e., protective direction [8,10]. Thus, our findings may point to a potential link between AATD mutations and exacerbations, although this association does not appear consistent with previous reports. It is true that none of the previous works, nor ours, have included cases with SERPINA1 PI*ZZ genotypes, so this would be the question that would still remain open about the relationship between CF and AATD. Interestingly, a recent study that conducted whole-genome sequencing in pwCF did not identify the SERPINA1 gene as significantly associated with the disease [33].

The measurement of CRP alongside AAT in the screening for AATD is routinely recommended. This recommendation is based on the potential elevation of AAT due to inflammatory stimuli, as AAT functions as an acute phase reactant [34]. Among all acute phase reactants, CRP is the most extensively studied in the literature. In the context of bronchiectasis, this is particularly significant because chronic inflammation and chronic bronchial infection create a persistent inflammatory state that could alter the levels of acute phase reactants. Notably, our study indicates that while the level of AAT is modified, the frequency of mutations remains equally distributed.

## 5. Conclusions

In conclusion, our results define the frequency of SERPINA1 gene mutations in adult and pediatric individuals with CF, consolidating prevalence estimates in this population and providing a practical cut-off point for AATD screening. AATD mutations are prevalent in CF and may impact certain clinical outcomes. If systematic screening is planned, we recommend considering the proposed cut-off points to select the population for genetic studies. Systematic screening could facilitate earlier detection, refined prognostic assessment, and more personalized management. Future research should aim to validate these cut-off values in larger multicenter cohorts, explore their utility in longitudinal follow-up, and determine the extent to which AATD status modifies disease progression or therapeutic responses.

## Figures and Tables

**Table 1:** Baseline characteristics of the patients.

Variable	No AATD (n = 311)	Any AATD Mutation (n = 58)	*p* Value *
Age (years)	22.9 (14.2)	24.3 (16.9)	0.243
Pediatric (<18 y.) cases (n)	129 (41.5)	22 (37.9)	0.614
Gender (male)	155 (49.8)	21 (36.2)	0.056
Never smokers (n)	293 (94.2)	56 (96.6)	0.350
Tobacco history (pack-years)	11.7 (9.7)	20.0 (14.1)	0.143
Age upon CF diagnosis (years)	6.3 (11.9)	7.8 (14.1)	0.187
CF mutations (n):			0.073
F508del homozygous	85 (27.4)	14 (24.1)	
F508del heterozygous	156 (50.3)	23 (39.7)	
Other	69 (22.3)	21 (36.2)	
Body mass index (kg/m^2^)	20.7 (4.3)	21.0 (4.1)	0.315
FVC (liters)	3.1 (1.2)	2.8 (1.0)	0.084
FVC (%)	90.9 (18.6)	85.6 (31.9)	0.063
FEV_1_ (liters)	2.2 (0.9)	2.0 (0.7)	0.043
FEV_1_ (%)	77.3 (24.6)	74.0 (24.6)	0.378
Chronic bronchial infection (n)	188 (60.3)	32 (56.1)	0.453
CFTR modulators (n)	161 (51.8)	19 (32.8)	0.008

Data expressed as mean (standard deviation) or as absolute (relative) frequencies depending on the nature of the variable. * *p* value calculated by chi-square or unpaired *t* tests, according to the nature of the variable. CF: cystic fibrosis. CFTR: cystic fibrosis transmembrane conductance regulator. FVC: forced vital capacity. FEV_1_: forced expiratory volume in 1 s.

**Table 2:** General view of studies of AATD prevalence in CF.

Study	Population	PI*MS	PI*MZ	PI*SS	PI*SZ	Other
Döring G et al. Pediatr Pulmonol 1994 [8]	215 pwCF	17 (7.9%)	10 (4.6)	1 (0.4%)	–	–
Mahadeva R et al. Thorax 1998 [9] *	79 pwCF	4 (5.0%)	1 (1.2%)	–	–	–
Mahadeva R et al. Eur Respir J 1998 [10]	147 pwCF	16 (10.1%)	3 (1.9%)	1 (0.6%)	–	–
Meyer P et al. Clin Genet 2002 [11]	269 pwCF	16 (6%)	5 (1.9%)	–	–	–
Frangolias DD et al. Am J Respir Cell Mol Biol 2003 [12]	716 pwCF	69 (9.6%)	18 (2.5%)	13 (1.8%)	–	–
Amati F et al. Biomedicines 2022 [28]	173 pwCF	9 (5.2%)	–	–	–	–
Current study	369 pwCF	47 (12.7%)	5 (1.4%)	3 (0.8%)	2 (0.5%)	PI*M/P_lowell_ 1 (0.3%)
Pooled analysis	1889 pwCF *	174 (9.2%)	42 (2.2%)	17 (0.8%)	2 (0.1%)	1 (0.05%)

* The study by Mahadeva R et al. Thorax 1998 [9] was not included in the pooled analysis, as those patients were already incorporated in the subsequent study from the same group [10].

## Data Availability

The data supporting this research are available upon reasonable request for research purposes.

## Supplementary Materials

The following supporting information can be downloaded at: https://www.mdpi.com/article/10.3390/jcm14196789/s1, Figure S1: Kaplan–Meier survival curves depicting the association between exacerbations and AATD-related mutations; Table S1: Multivariate analysis of FEV_1_ on the index date between cases carrying any SERPINA1 mutation for (a) all mutations and (b) excluding PI*MS cases; Table S2: Biochemical and hematological parameters between patients with and without mutations associated with AATD.

## Abbreviations

The following abbreviations are used in this manuscript:

AAT	Alpha1 antitrypsin
AATD	Alpha1 antitrypsin deficiency
CF	Cystic Fibrosis
CRP	C-reactive protein
FADO-CF	Finding AAT Deficiency in Obstructive Lung Diseases: Cystic Fibrosis
FEV_1_	Forced expiratory volume in one second
pwCF	people with CF
ROC	receiver operating characteristic
