# Supplementary material for: Evaluation of Alpha1 Antitrypsin Deficiency-Associated Mutations in People with Cystic Fibrosis
Source: J Clin Med. 2025 Sep 25;14(19):6789. doi: 10.3390/jcm14196789 (PMC12524791; doi:10.3390/jcm14196789)

**ONLINE SUPPLEMENT**

**Evaluation of alpha1 antitrypsin deficiency associated mutations in people with cystic fibrosis.**

**Authors:** Jose Luis Lopez-Campos (1,2), Pedro García Tamayo (1), Maria Victoria Girón (3), Isabel Delgado-Pecellín (2,4), Gabriel Olveira (5,6), Laura Carrasco (1,2), Rocío Reinoso-Arija (1), Casilda Olveira (3), Esther Quintana-Gallego (1,2)

**Institutions:**

1. Unidad Médico-Quirúrgica de Enfermedades Respiratorias. Instituto de Biomedicina de Sevilla (IBiS). Hospital Universitario Virgen del Rocío/Universidad de Sevilla, Spain.
2. Centro de Investigación Biomédica en Red de Enfermedades Respiratorias (CIBERES). Instituto de Salud Carlos III, Madrid, Spain
3. Servicio de Neumología. Hospital Regional Universitario de Málaga. Departamento de Medicina y Dermatología. Universidad de Málaga. Instituto de Investigación Biomédica de Málaga (IBIMA)- Plataforma Bionand, Málaga, Spain.
4. Servicio de Pediatría. Hospital Universitario Virgen del Rocío, Seville, Spain
5. Servicio de Endocrinología y Nutrición. Hospital Regional Universitario de Málaga. Departamento de Medicina y Dermatología. Universidad de Málaga. Instituto de Investigación Biomédica de Málaga (IBIMA)- Plataforma Bionand, Málaga, Spain.
6. Centro de Investigación Biomédica en Red de Diabetes y Enfermedades Metabólicas Asociadas (CIBERDEM). Instituto de Salud Carlos III, Madrid, Spain

**Correspondence:** José Luis Lopez-Campos. Hospital Universitario Virgen del Rocío. Avda. Manuel Siurot, s/n. 41013 Seville, Spain. Tel: +34 955013166. Email; [lcampos@separ.es](mailto:lcampos@separ.es)

Table S1. Multivariate analysis of FEV_1_ on the index date between cases carrying any SERPINA1 mutation for a) all mutations and b) excluding PI*MS cases.

a) All mutations (n=369)

| Variable | Crude | | Adjusted | |
| --- | --- | --- | --- | --- |
|  | OR | 95%CI | OR | 95%CI |
| FEV_1_ (%) | 0.995 | 0.983-1.007 | 0.994 | 0.981-1.008 |
| Gender (male) | 0.571 | 0.320-1.020 | 0.707 | 0.371-1.347 |
| Age (years) | 1.007 | 0.988-1.026 | 1.011 | 0.988-1.035 |
| F508del carrier | 0.504 | 0.277-0.918 | 0.589 | 0.290-1.196 |
| CFTR modulators | 0.454 | 0.251-0.820 | 0.627 | 0.306-1.286 |

FEV_1_ forced expiratory volume in one second. CFTR: Cystic Fibrosis Transmembrane Conductance Regulator. OR: odds ratio. CI: confidence interval.

b) Excluding PI*MS (n=322)

| Variable | Crude | | Adjusted | |
| --- | --- | --- | --- | --- |
|  | OR | 95%CI | OR | 95%CI |
| FEV_1_ (%) | 0.979 | 0.956-1.003 | 0.040 | 0.941-0.999 |
| Gender (male) | 2.684 | 0.699-10.305 | 2.880 | 0.703-11.798 |
| Age (years) | 1.001 | 0.959-1.044 | 0.956 | 0.895-1.022 |
| F508del carrier | 0.763 | 0.197-2.956 | 0.576 | 0.127-2.607 |
| CFTR modulators | 1.630 | 0.468-5.682 | 0.985 | 0.230-4.211 |

FEV_1_ forced expiratory volume in one second. CFTR: Cystic Fibrosis Transmembrane Conductance Regulator. OR: odds ratio. CI: confidence interval.

Table S2. Biochemical and hematological parameters between patients with and without mutations associated with AATD.

| Parameter | Without AATD mutations  (n=311) | With AATD mutations  (n=58) | P value * |
| --- | --- | --- | --- |
| Alpha1 antitrypsin (mg/dl) | 147.4 (32.2) | 122.2 (35.5) | < 0.001 |
| C-reactive protein (mg/l) | 5.3 (13.6) | 4.6 (9.1) | 0.364 |
| Erythrocyte sedimentation rate (mm/h) | 11.8 (28.6) | 11.4 (9.2) | 0.472 |
| Ferritin (ng/ml) | 76.3 (66.3) | 94.9 (95.4) | 0.143 |
| Fibrinogen (mg/dl) | 239.6 (153.9) | 230.5 (148.3) | 0.377 |
| Bilirubin total (mg/dl) | 0.5 (0.4) | 0.5 (0.3) | 0.180 |
| Alkaline phosphatase (U/l) | 183.0 (264.2) | 175.9 (141.2) | 0.423 |
| Aspartate aminotransferase (U/l) | 32.8 (35.8) | 31.6 (19.4) | 0.410 |
| Alanine aminotransferase (U/l) | 32.0 (54.0) | 31.0 (24.8) | 0.444 |
| Albumin (g/dl) | 4.1 (0.4) | 4.1 (0.5) | 0.783 |
| Prothrombin time (s) | 2.5 (12.0) | 1.0 (0.08) | 0.190 |
| Platelet count (10^3^ cells/µl) | 305.6 (102.3) | 313.5 (118.4) | 0.301 |

Data expressed ad mean (standard deviation). * p value estimated with unpaired Student t test.

Figure S1. Kaplan–Meier survival curves depicting the association between exacerbations and AATD-related mutations:

a) Time to first mild exacerbation in individuals carrying any AATD mutation.


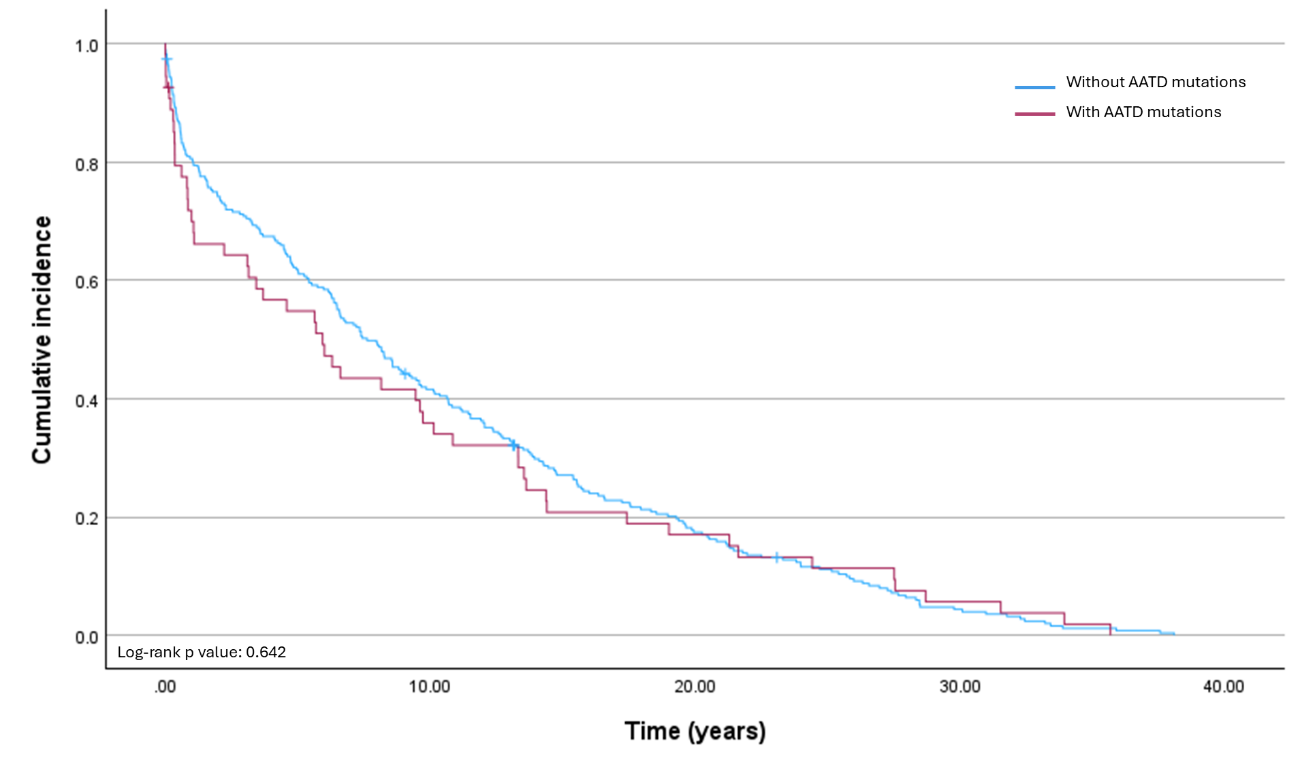


b) Time to first severe exacerbation in individuals carrying any AATD mutation.


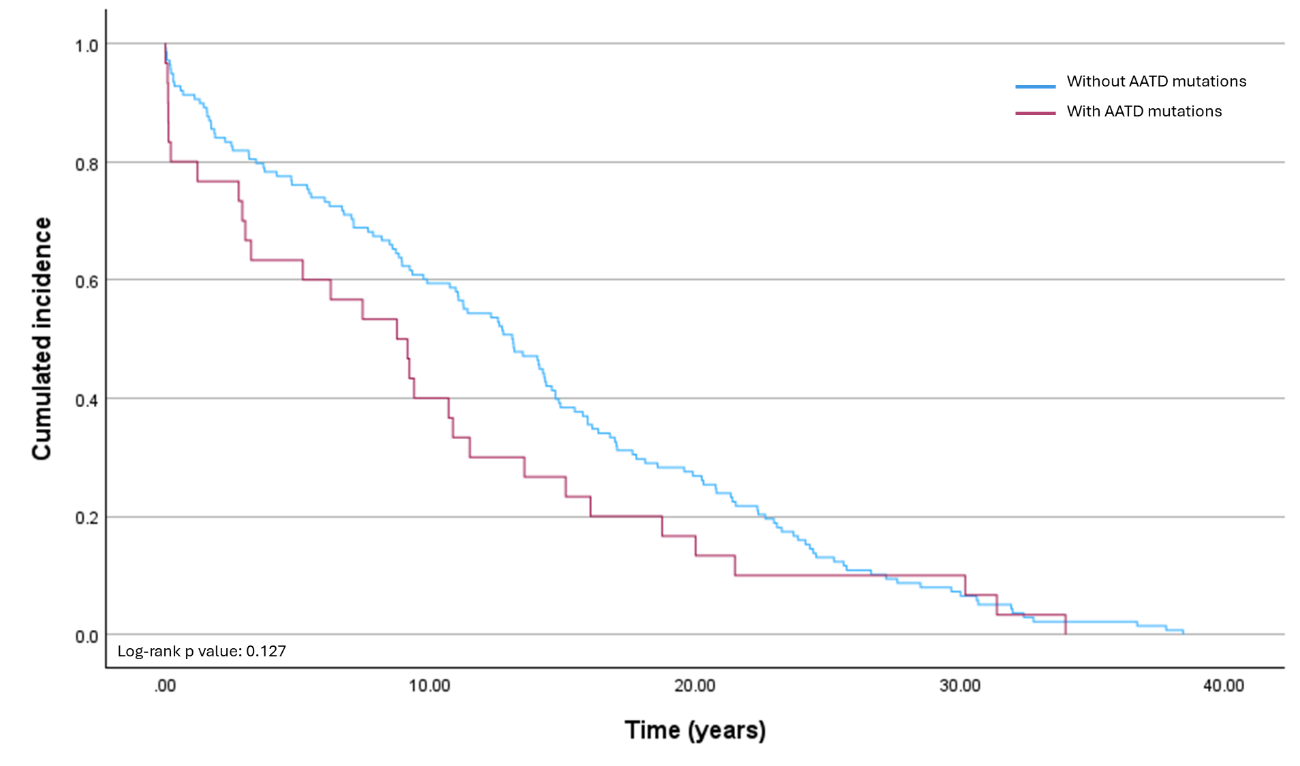


c) Time to first mild exacerbation, excluding PIMS carriers.


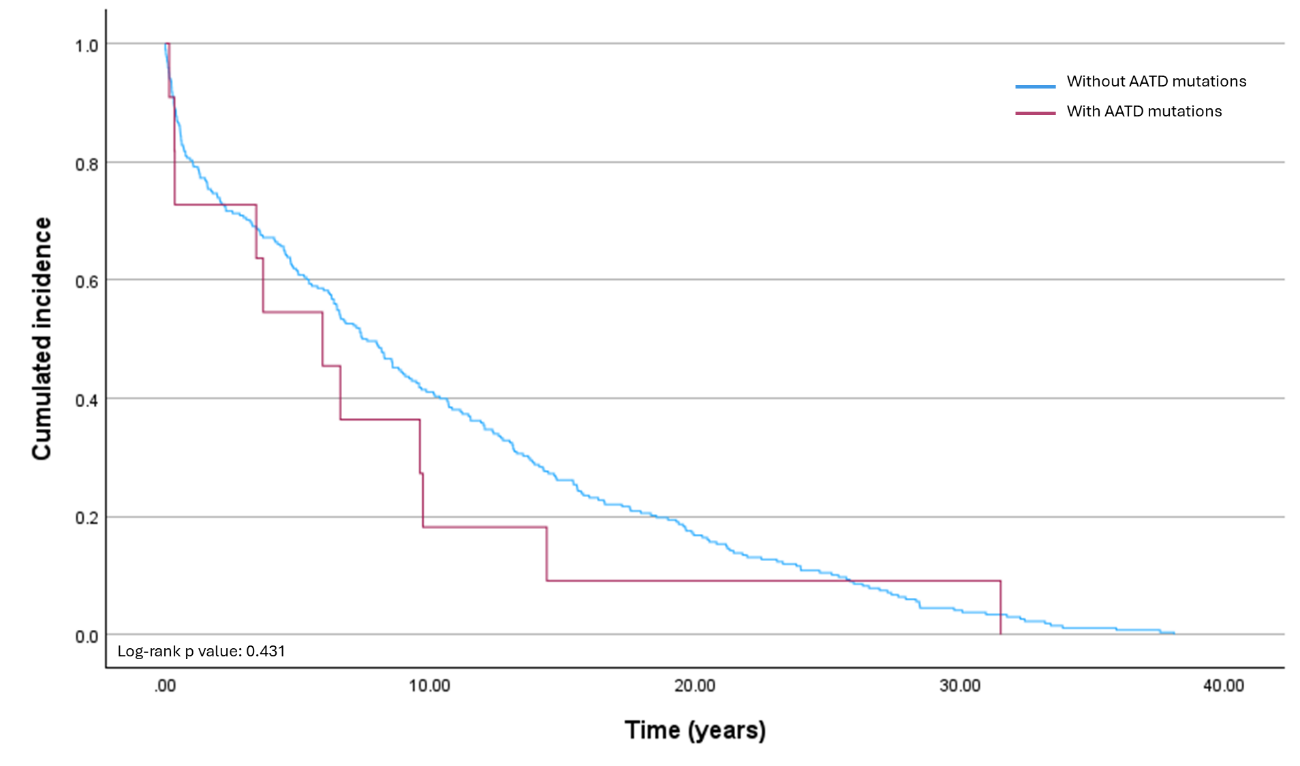


d) Time to first severe exacerbation, excluding PIMS carriers.


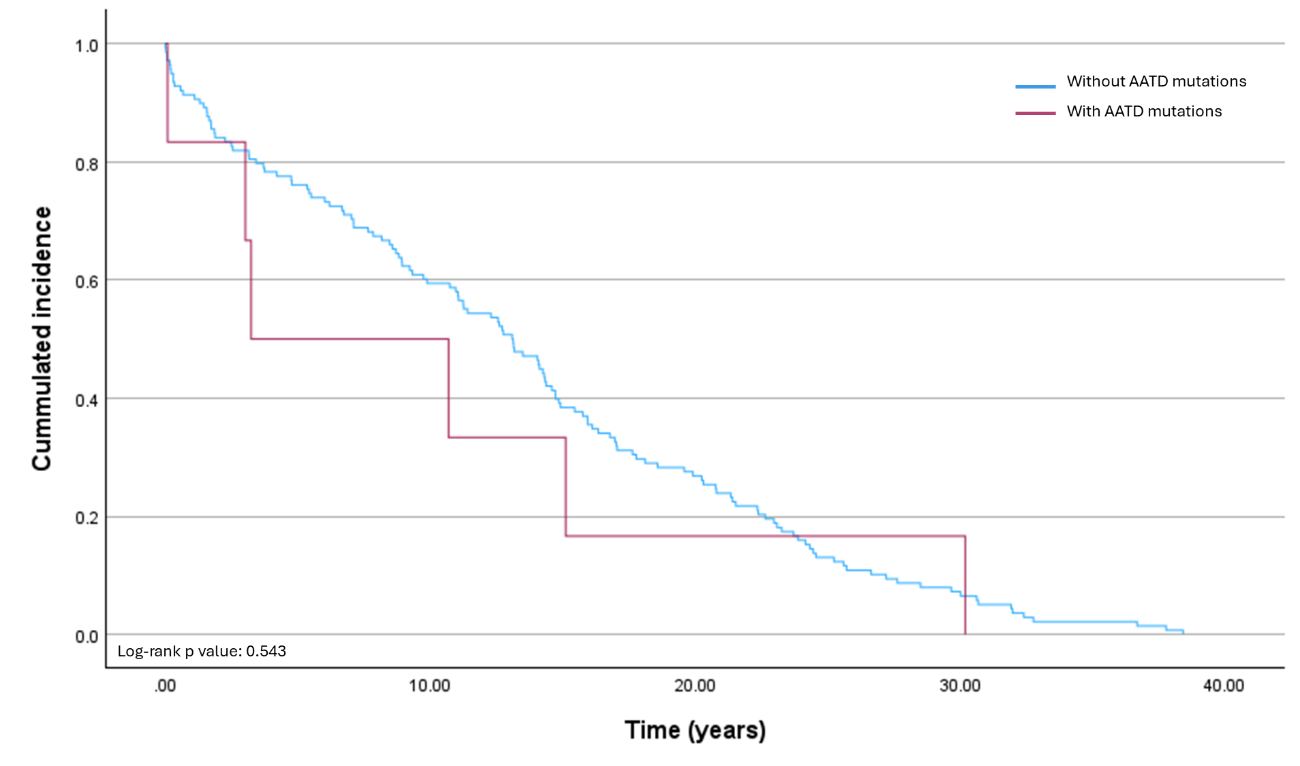

Supplement: Supplementary file 1 [file jcm-14-06789-s001.zip › jcm-3798105-supplementary/jcm-3798105-supplementary.docx]
