# Supplementary material for: Evaluation of Alpha1 Antitrypsin Deficiency-Associated Mutations in People with Cystic Fibrosis
Source: J Clin Med. 2025 Sep 25;14(19):6789. doi: 10.3390/jcm14196789 (PMC12524791; doi:10.3390/jcm14196789)

ONLINE SUPPLEMENT

Evaluation of alpha1 antitrypsin deficiency associated mutations in people with cystic fibrosis.

Authors: Jose Luis Lopez-Campos (1,2), Pedro García Tamayo (1), Maria Victoria Girón (3), Isabel Delgado-Pecellín (2,4), Gabriel Olveira (5,6), Laura Carrasco (1,2), Rocío Reinoso-Arija (1), Casilda Olveira (3), Esther Quintana-Gallego (1,2)

Institutions:

(1) Unidad Médico-Quirúrgica de Enfermedades Respiratorias. Instituto de Biomedicina de Sevilla (IBiS). Hospital Universitario Virgen del Rocío/Universidad de Sevilla, Spain.
(2) Centro de Investigación Biomédica en Red de Enfermedades Respiratorias (CIBERES). Instituto de Salud Carlos III, Madrid, Spain
(3) Servicio de Neumología. Hospital Regional Universitario de Málaga. Departamento de Medicina y Dermatología. Universidad de Málaga. Instituto de Investigación Biomédica de Málaga (IBIMA)- Plataforma Bionand, Málaga, Spain.
(4) Servicio de Pediatría. Hospital Universitario Virgen del Rocío, Seville, Spain
(5) Servicio de Endocrinología y Nutrición. Hospital Regional Universitario de Málaga. Departamento de Medicina y Dermatología. Universidad de Málaga. Instituto de Investigación Biomédica de Málaga (IBIMA)- Plataforma Bionand, Málaga, Spain.
(6) Centro de Investigación Biomédica en Red de Diabetes y Enfermedades Metabólicas Asociadas (CIBERDEM). Instituto de Salud Carlos III, Madrid, Spain

Correspondence: José Luis Lopez-Campos. Hospital Universitario Virgen del Rocío. Avda. Manuel Siurot, s/n. 41013 Seville, Spain. Tel: +34 955013166. Email; lcampos@separ.es

Table S1. Multivariate analysis of FEV_1 on the index date between cases carrying any SERPINA1 mutation for a) all mutations and b) excluding PI*MS cases.

a) All mutations (n=369)

Variable	Crude		Adjusted	
	OR	95%CI	OR	95%CI
FEV_1 (%)	0.995	0.983-1.007	0.994	0.981-1.008
Gender (male)	0.571	0.320-1.020	0.707	0.371-1.347
Age (years)	1.007	0.988-1.026	1.011	0.988-1.035
F508del carrier	0.504	0.277-0.918	0.589	0.290-1.196
CFTR modulators	0.454	0.251-0.820	0.627	0.306-1.286

FEV_1 forced expiratory volume in one second. CFTR: Cystic Fibrosis Transmembrane Conductance Regulator. OR: odds ratio. CI: confidence interval.

b) Excluding PI*MS (n=322)

Variable	Crude		Adjusted	
	OR	95%CI	OR	95%CI
FEV_1 (%)	0.979	0.956-1.003	0.040	0.941-0.999
Gender (male)	2.684	0.699-10.305	2.880	0.703-11.798
Age (years)	1.001	0.959-1.044	0.956	0.895-1.022
F508del carrier	0.763	0.197-2.956	0.576	0.127-2.607
CFTR modulators	1.630	0.468-5.682	0.985	0.230-4.211

FEV_1 forced expiratory volume in one second. CFTR: Cystic Fibrosis Transmembrane Conductance Regulator. OR: odds ratio. CI: confidence interval.

Table S2. Biochemical and hematological parameters between patients with and without mutations associated with AATD.

Parameter	Without AATD mutations (n=311)	With AATD mutations (n=58)	P value *
Alpha1 antitrypsin (mg/dl)	147.4 (32.2)	122.2 (35.5)	< 0.001
C-reactive protein (mg/l)	5.3 (13.6)	4.6 (9.1)	0.364
Erythrocyte sedimentation rate (mm/h)	11.8 (28.6)	11.4 (9.2)	0.472
Ferritin (ng/ml)	76.3 (66.3)	94.9 (95.4)	0.143
Fibrinogen (mg/dl)	239.6 (153.9)	230.5 (148.3)	0.377
Bilirubin total (mg/dl)	0.5 (0.4)	0.5 (0.3)	0.180
Alkaline phosphatase (U/l)	183.0 (264.2)	175.9 (141.2)	0.423
Aspartate aminotransferase (U/l)	32.8 (35.8)	31.6 (19.4)	0.410
Alanine aminotransferase (U/l)	32.0 (54.0)	31.0 (24.8)	0.444
Albumin (g/dl)	4.1 (0.4)	4.1 (0.5)	0.783
Prothrombin time (s)	2.5 (12.0)	1.0 (0.08)	0.190
Platelet count (10^3 cells/µl)	305.6 (102.3)	313.5 (118.4)	0.301

Data expressed ad mean (standard deviation). * p value estimated with unpaired Student t test.

Figure S1. Kaplan–Meier survival curves depicting the association between exacerbations and AATD-related mutations:

a) Time to first mild exacerbation in individuals carrying any AATD mutation.

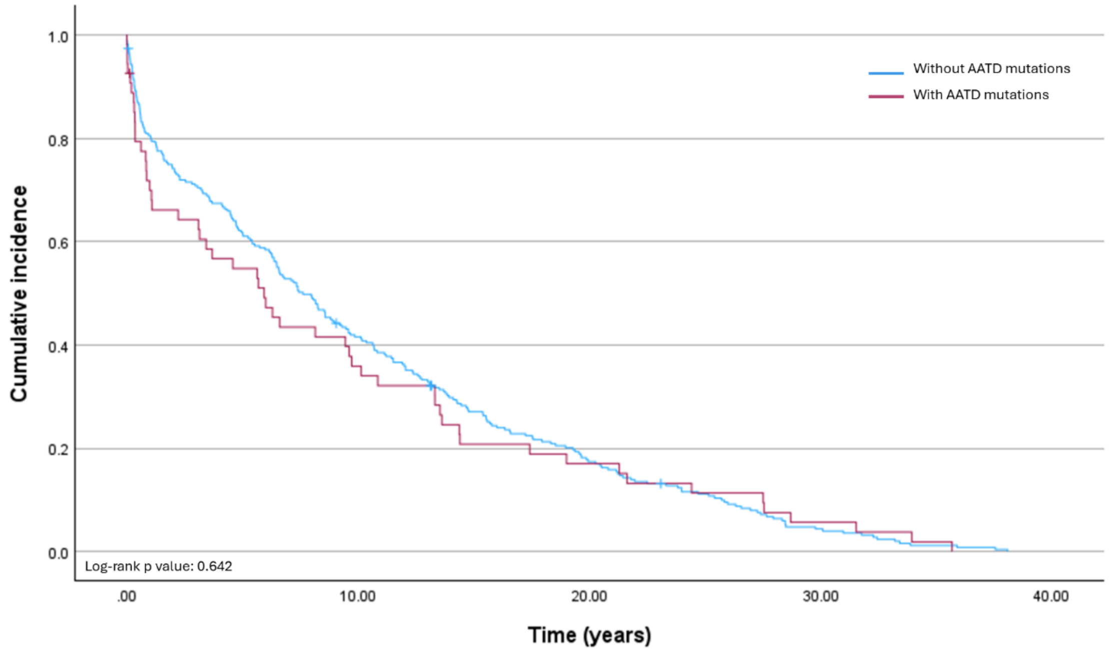

b) Time to first severe exacerbation in individuals carrying any AATD mutation.

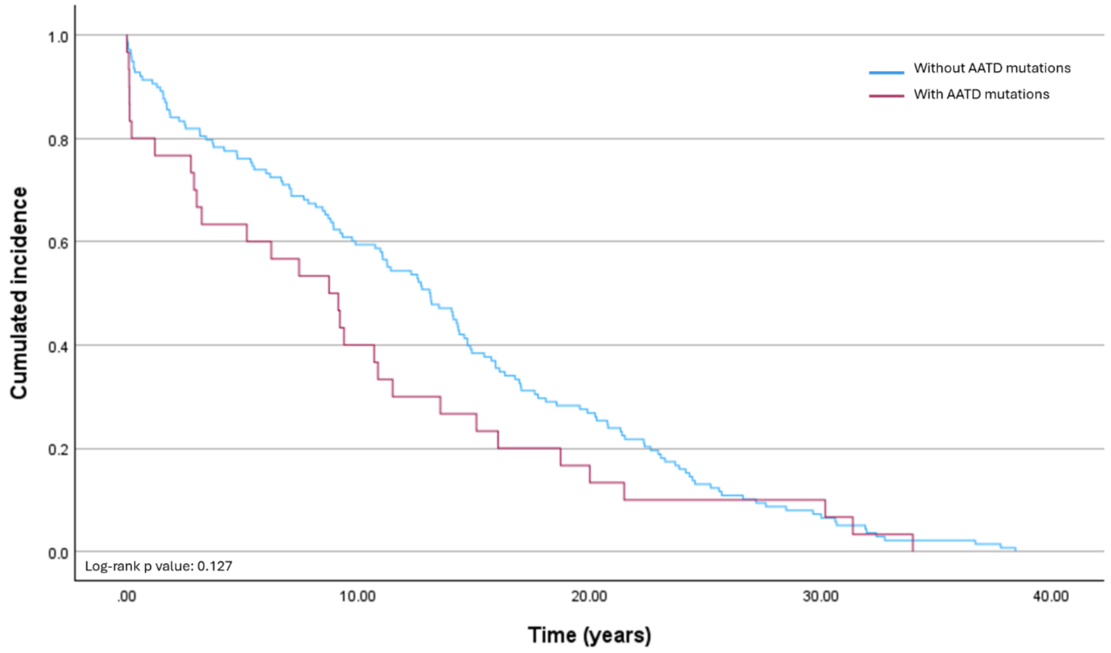

c) Time to first mild exacerbation, excluding PIMS carriers.

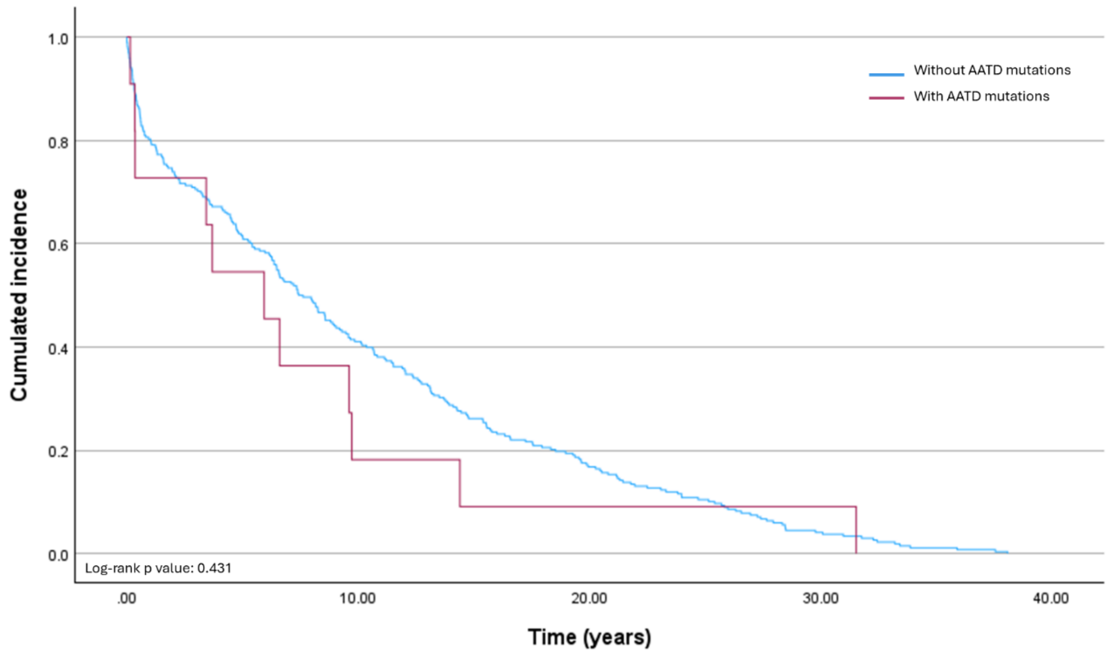

d) Time to first severe exacerbation, excluding PIMS carriers.

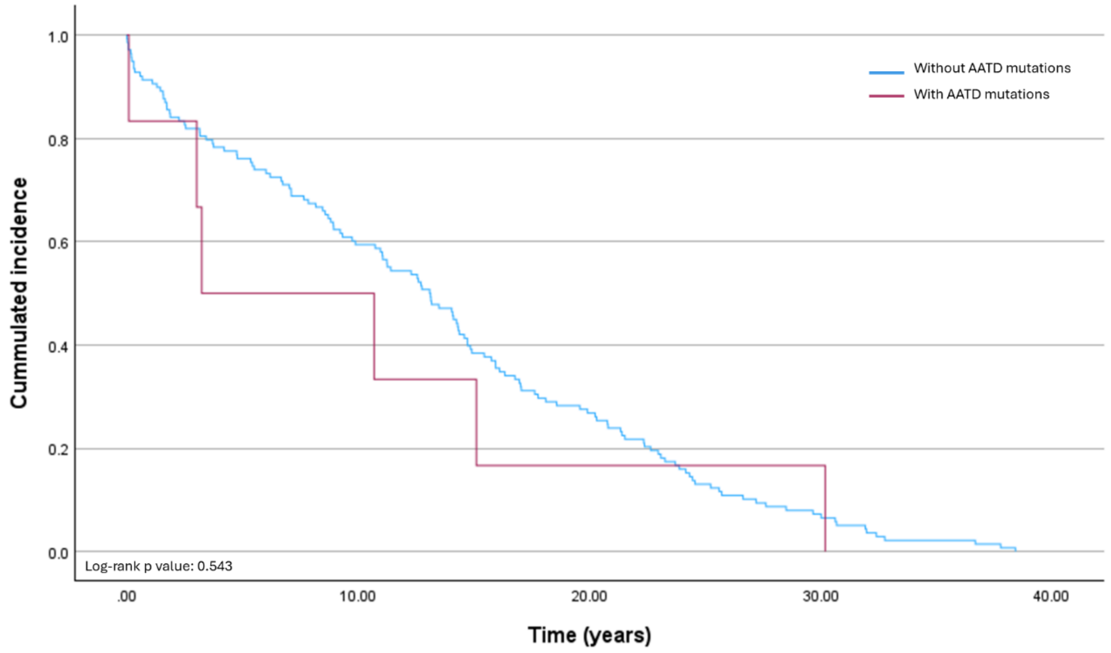

Supplement: Supplementary file 1 [file jcm-14-06789-s001.zip › jcm-3798105-supplementary/jcm-3798105-supplementary.pdf]
